# Smokeless tobacco consumption among women of reproductive age: a systematic review and meta-analysis

**DOI:** 10.1186/s12889-024-18840-z

**Published:** 2024-05-20

**Authors:** Ramaiah Itumalla, Mahalaqua Nazli Khatib, Shilpa Gaidhane, Quazi Syed Zahiruddin, Abhay M Gaidhane, Ahmad Neyazi, Ahmad Farshid Hassam, Prakasini Satapathy, Sarvesh Rustagi, Neelima Kukreti, Bijaya Kumar Padhi

**Affiliations:** 1 School of Management, The Apollo University, Chittoor, 517127 Andhra Pradesh India; 2 Division of Evidence Synthesis, Global Consortium of Public Health and Research, Datta Meghe Institute of Higher Education, Wardha, India; 3 One Health Centre (COHERD), Jawaharlal Nehru Medical College, Datta Meghe Institute of Higher Education, Wardha, India; 4 South Asia Infant Feeding Research Network (SAIFRN), Division of Evidence Synthesis, Global Consortium of Public Health and Research, Datta Meghe Institute of Higher Education, Wardha, India; 5 Jawaharlal Nehru Medical College, and Global Health Academy, School of Epidemiology and Public Health, Datta Meghe Institute of Higher Education, Wardha, India; 6 Afghanistan Center for Epidemiological Studies, Herat, Afghanistan; 7 Scientific Affairs, Herat Regional Hospital, Herat, Afghanistan; 8 Center for Global Health Research, Saveetha Institute of Medical and Technical Sciences, Saveetha Medical College and Hospital, Saveetha University, Chennai, India; 9 Medical Laboratories Techniques Department, AL-Mustaqbal University, Hillah, Babil, 51001 Iraq; 10 School of Applied and Life Sciences, Uttaranchal University, Dehradun, Uttarakhand India; 11 School of Pharmacy, Graphic Era Hill University, Dehradun, India; 12 Department of Community Medicine, School of Public Health, Postgraduate Institute of Medical Education and Research, Chandigarh, 160012 India

**Keywords:** Smokeless tobacco, Women of reproductive age, Meta-analysis, Public health, Chewing tobacco, Sustainable development goals

## Abstract

Smokeless tobacco (SLT) poses many negative health impacts. Despite its longstanding presence in societies across the world, the health implications of SLT have only been rigorously studied in recent decades. This systematic review and meta-analysis aimed to consolidate existing research to provide a comprehensive understanding of the global prevalence of SLT use among women of reproductive age. Relevant articles were extracted from databases such as PubMed, EMBASE, Web of Science, and Scopus from their inception until November 11, 2023. Observational studies reporting the number of SLT users among women of reproductive age were included. The quality of the studies was assessed using the Joanna Briggs Institute (JBI) tool. The meta-analysis used a random-effects model to determine SLT prevalence, supported by statistical tools like forest plots, I^2^ statistics, and sensitivity analyses to ensure the accuracy and comprehensiveness of the results. All statistical analyses were performed in R version 4.3. From 10 studies involving 2,053,667 participants, a pooled prevalence for SLT use among women of reproductive age was found to be 9.3% (95% CI: 0.038 to 0.21), with significant heterogeneity among studies (I^2^ = 100%). Publication bias was suspected among the studies. Sensitivity analysis and subgroup analysis couldn’t resolve the heterogeneity. Our analysis shows a significant prevalence of SLT use in women of reproductive age, especially in low socioeconomic and developing countries like India, Pakistan, and Nepal. For women of reproductive age, the use of smokeless tobacco (SLT) can lead to infertility, pregnancy complications, and adverse fetal outcomes, including low birth weight and preterm birth. The results highlight the necessity for specific public health measures and policy changes to decrease SLT consumption among reproductive-age women. Further studies are needed to investigate the reasons behind SLT usage in this group and to assess the impact of intervention strategies, to guide more effective public health initiatives and policies.

**Supplementary Information:**

The online version contains supplementary material available at 10.1186/s12889-024-18840-z.

## Introduction

Tobacco consumption represents a major health concern due to its strong association with a range of chronic diseases [1]. Over two-thirds of fatalities in developing nations are attributed to non-communicable diseases (NCDs), and tobacco use is a major preventable contributor to these deaths [2]. Despite numerous international policy initiatives, aimed at reducing tobacco consumption, such as the Framework Convention on Tobacco Control (FCTC) by the World Health Organization, the trend in tobacco use continues unabated [3]. By 2030, it has been estimated that tobacco use will result in ten million premature deaths per year [4]. Developing countries are disproportionately affected, contributing to nearly 70% of tobacco consumption and associated deaths [4, 5].

Tobacco products are categorized into smoking types, including cigarettes, cigars, and pipes, and smokeless forms, such as snus, chewing tobacco, and dissolvable tobacco, each contributing to the health epidemic [6–11]. Globally, smokeless tobacco (SLT) is consumed in various forms, such as chewing tobacco, snuff, and snus [12]. Historically, the use of SLT has deep roots in many cultures, often seen as a part of social rituals and traditional practices [13]. Despite the longstanding presence of SLT in societies across the world, its health implications have only been rigorously studied in recent decades. SLT is often misleadingly perceived as a safer alternative to smoked tobacco, which may also has contributed to its widespread use [14–16]. This misconception is particularly prevalent in regions like South Asia, parts of Europe, and North America. The cultural acceptance and traditional use of SLT in these regions complicate efforts to mitigate its health risks [17, 18]. Understanding the complexities of SLT consumption, its cultural ties, and perceived risks is crucial for comprehensive research, especially when focusing on specific demographics such as women of reproductive age [18].

Over 30 carcinogens were contained in SLT and those strongly associated with multiple types of cancers, including oral, oropharyngeal, oesophageal, and pancreatic cancer [19–25]. It also significantly contributes to cardiovascular diseases and hypertension [26]. The systemic impact of SLT extends beyond these direct health risks. It affects oral hygiene, leading to dental issues, and contributes to increased susceptibility to infections [27]. For women of reproductive age, the use of SLT introduces additional concerns, such as infertility [28], complications during pregnancy, and adverse fetal outcomes, like low birth weight and preterm birth [29].

The prevalence of SLT use among women of reproductive age (15 to 49 years) is a critical public health issue. Women in this demographic are particularly vulnerable due to potential adverse effects on both themselves and their offspring. In some cultures, SLT use among women is a taboo, leading to underreporting and lack of visibility in public health discourses. In others, SLT is used as a traditional remedy for ailments like toothache and nausea during pregnancy, exacerbating its use among women of reproductive age [30–32].

Several systematic reviews have focused on different aspects of SLT, covering its adverse outcomes and risk factors [12, 33–36]. Women of reproductive age represent a crucial demographic due to their potential impact on both maternal and child health. SLT use in this group can have significant adverse outcomes, including impacts on pregnancy outcomes, fetal development, and increased risk of developing non-communicable diseases. Understanding the prevalence of SLT use among these women is vital for targeted public health interventions and policies. While individual primary studies have been published on the prevalence of SLT use among women of reproductive age, to date, no systematic review has assessed the overall prevalence of SLT in this specific population [33–36]. This review aims to guide public health strategies, inform policies, and spur further research to decrease smokeless tobacco use among reproductive-aged women, aligning with the sustainable development goals (SDG-3) to ensure healthy lives and promote well-being for all ages, thereby enhancing maternal and child health outcomes.

## Methods

This systematic review was conducted according to Preferred Reporting Items for Systematic Reviews and Meta-Analyses (PRISMA) guidelines [37] (Table S1). A protocol has been prospectively registered in the PROSPERO: CRD42023482095.

### Selection criteria

Original research conducted among women of reproductive age 15 to 49, published in peer-reviewed journals was considered for inclusion. Observational studies like surveys, cross-sectional studies, and longitudinal studies were included. Studies which reported the proportion of women consumed SLT were included. Studies that reported SLT use among the general population or men were excluded. Reviews, case reports, case series, non-human studies were also excluded. Articles available in the English language were considered (Table S2).

### Literature search and screening

A literature search was conducted among databases like PubMed, EMBASE, Web of Science, and Scopus since inception to November 11, 2023. Keywords and MeSH terms related to SLT, reproductive women were used to search. No restrictions were placed on the search regarding the type of article, year of publication, or language (refer Table S3).

The screening process was performed in two steps: title and abstract screening followed by full-text screening. A semi-automated software (Nested Knowledge, USA) was used for de-duplication and screening. Two reviewers (RI and MNK) performed screening independently. A third reviewer (BKP) was consulted to resolve discrepancies regarding the inclusion of articles.

### Data extraction

Data extraction was performed by two reviewers (RI and MNK), followed by a double-check conducted by a third reviewer (QSZ) to ensure accuracy and consistency. For each included article, the data extracted included the author’s name, year of publication, study design, country, age of participants, sample size, and the number of SLT users. This comprehensive approach allowed for a detailed analysis of the studies’ key characteristics and demographic information, providing a robust foundation for the systematic review.

To assess the quality of each included study, a meticulous quality assessment was performed using the Joanna Briggs Institute (JBI) tool for prevalence studies [38]. The JBI assessment tool for prevalence studies covers various aspects, including the representativeness of the sample, the appropriateness of the study’s methodology, the validity and reliability of the measures used, and the adequacy of response rates.

### Statistical analysis

A meta-analysis was conducted to determine the prevalence of SLT use among women of reproductive age, using a random-effects model to pool the number of SLT users and the total sample size of women in this age group. A forest plot was generated for visualization, and the I^2^ statistic was employed to assess study heterogeneity, indicating the percentage of variation due to heterogeneity rather than chance, with values ranging from 0 to 100% [39]. The analysis also included a 95% prediction interval and the tau-squared value for a deeper understanding of between-study variance [40, 41]. Subgroup analysis based on country and a leave-one-out sensitivity analysis were performed to gauge the influence of individual studies on overall results. Doi plot with LFK index was used to determine publication bias [42, 43]. Significance level was set at a p-value below 0.05, and all statistical analyses were conducted using R software, version 4.3 [44].

## Results

### Literature search

**Fig. 1:**
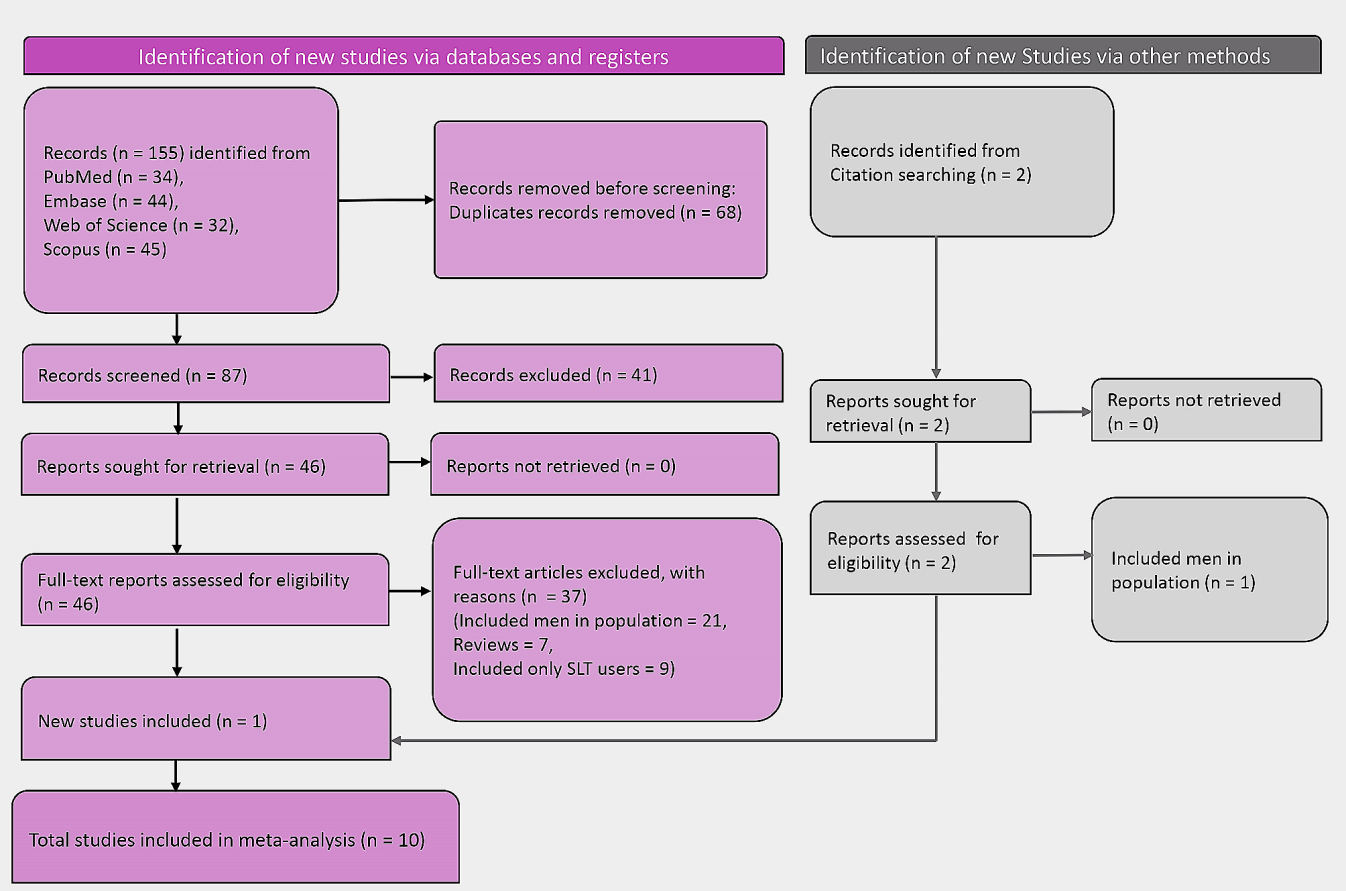
PRISMA flow diagram showing the screening and selection process

A total of 310 records were identified from multiple databases. Of these, 68 were duplicates. A total of 87 articles were subjected to screening, of which 41 were excluded. Subsequently, 46 articles were assessed for eligibility. Out of these, 9 articles were found eligible. Additionally, 2 articles were identified from a citation search, among which 1 article was found to be eligible. Finally, a total of 10 studies were included in this review. Figure 1 depicts the process of screening and selection of studies.

### Characteristics of included studies

Table 1 presents a summary of the features of the studies that were included. Out of the 10 studies, one was retrospective in design, while the rest were cross-sectional. The majority of these What studies were conducted in Southeast Asian regions (*n* = 7), specifically in countries like India (*n* = 5), Pakistan (*n* = 1), and Nepal (*n* = 1), reflecting a geographical focus on this area. One study included data from multiple countries. One study was available from Sweden. The age range of participants varied from 15 to 49 years, covering a wide spectrum of the female reproductive age group. The quality of the studies was found to be moderate to high, as assessed by the JBI tool (referenced in Table S4).

**Table 1:** Characteristics of the included studies

Study ID	Study design	Country	Age	Total sample	SLT users
Goyal et al. 2022 [45]	Cross-sectional	India	15–44	26,700	2612
Khan et al. 2015 [51]	Cross-sectional	Pakistan	15–49	12,995	277
Mishra et al. 2022 [46]	Retrospective observational	India	15–49	699,686	38,233
Mohandas et al. 2019 [47]	Cross-sectional	India	15–49	223	110
Muhammad et al. 2022 [50]	Cross-sectional	Pakistan	18–49	557	218
Niraula et al. 2004 [52]	Cross-sectional	Nepal	15–49	1902	264.00
Rolandsson et al. 2014 [54]	Cross-sectional	Sweden	15–24	784	39
Shukla et al. 2021 [53]	Cross-sectional	Multiple countries	15–49	1,230,262	10,580
Singh et al. 2022 [48]	Cross-sectional	India	15–49	79,729	7,499
Yuvaraj et al. 2020 [49]	Cross-sectional	India	15–49	829	148

### Meta-analysis

**Fig. 2:**
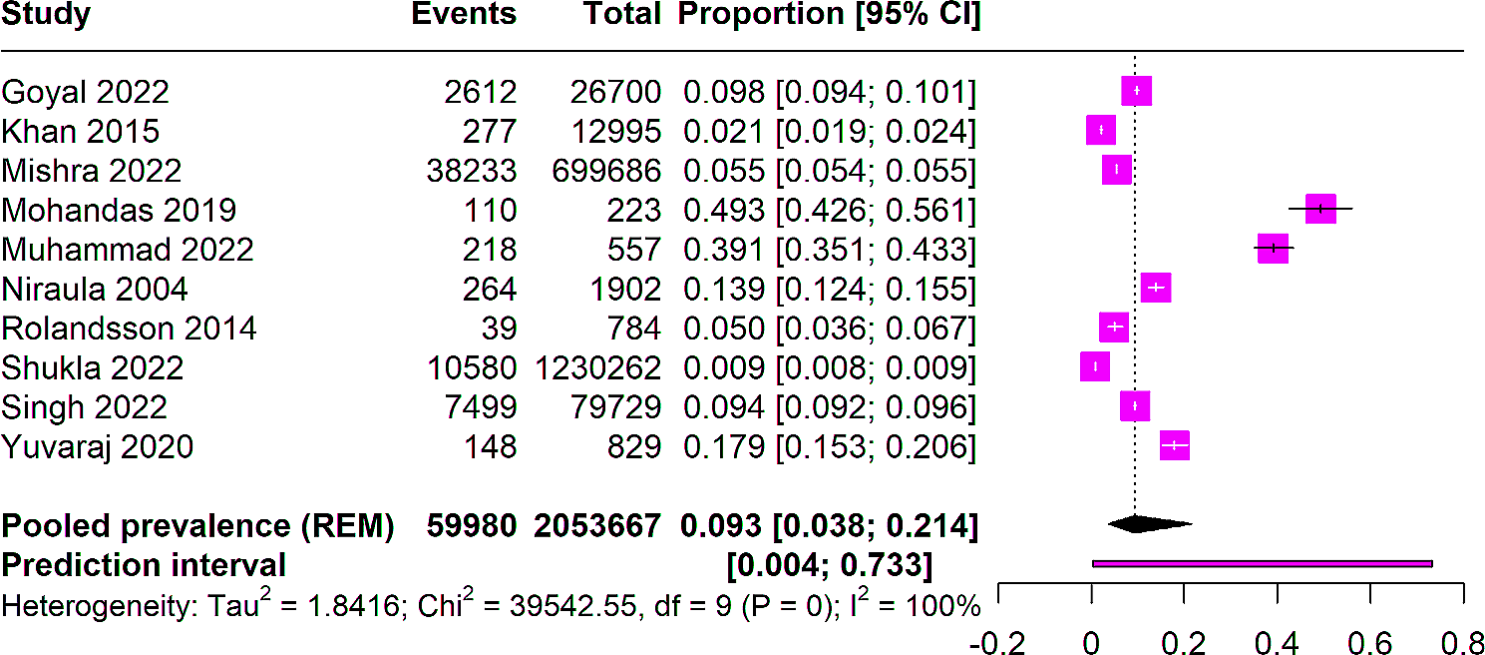
Forest plot depecting the pooled prevalence of SLT use among women of reproductive age

From 10 studies involving 2,053,667 participants, a pooled prevalence for SLT use among women of reproductive age was found to be 9.3% (95% CI: 0.038 to 0.21), with significant heterogeneity among studies (I^2^ = 100%). A prediction interval of 0.004 to 0.73 was observed. Figure 2 illustrates the forest plot. This indicates that, on average, about 9.3% of women of reproductive age in the studied populations use SLT. This high level of variability indicates that the prevalence rates of SLT use among women of reproductive age may vary widely across different studies and populations. The wide prediction interval ranging from 0.4 to 73% suggests that in a similar future study, the prevalence of SLT use among women of reproductive age could fall anywhere within this wide range. This reflects the significant variation in SLT use prevalence that could be expected across different settings and populations.

**Fig. 3:**
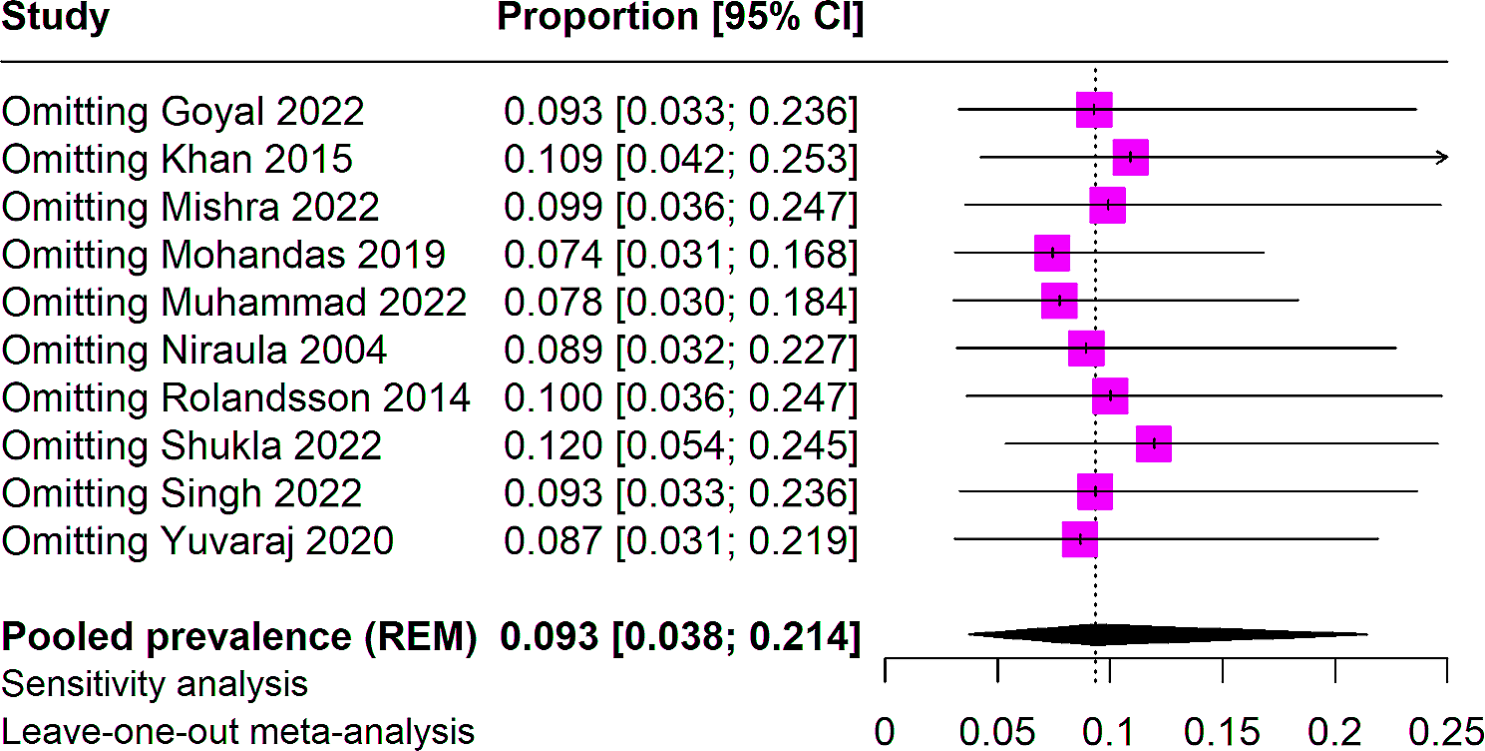
Leave one out analysis of pooled prevalence

The subgroup analysis based on country revealed that 5 studies from India [45–49] with 807,167 participants showed a prevalence of 14% (95% CI: 0.048 to 0.35) with I^2^ = 100%. Two studies from Pakistan [50, 51] revealed a similar prevalence of 10% (95% CI: 0.00 to 1.00). Nepal showed a prevalence of 13% (95% CI: 0.12 to 0.15) involving 1,902 participants from a single study [52]. In the study which included multiple countries [53], the prevalence was found to be 0.9% (95% CI: 0.008 to 0.009), involving 1,230,262 participants (Fig. 3).

### Sensitivity analysis and publication bias

**Fig. 4:**
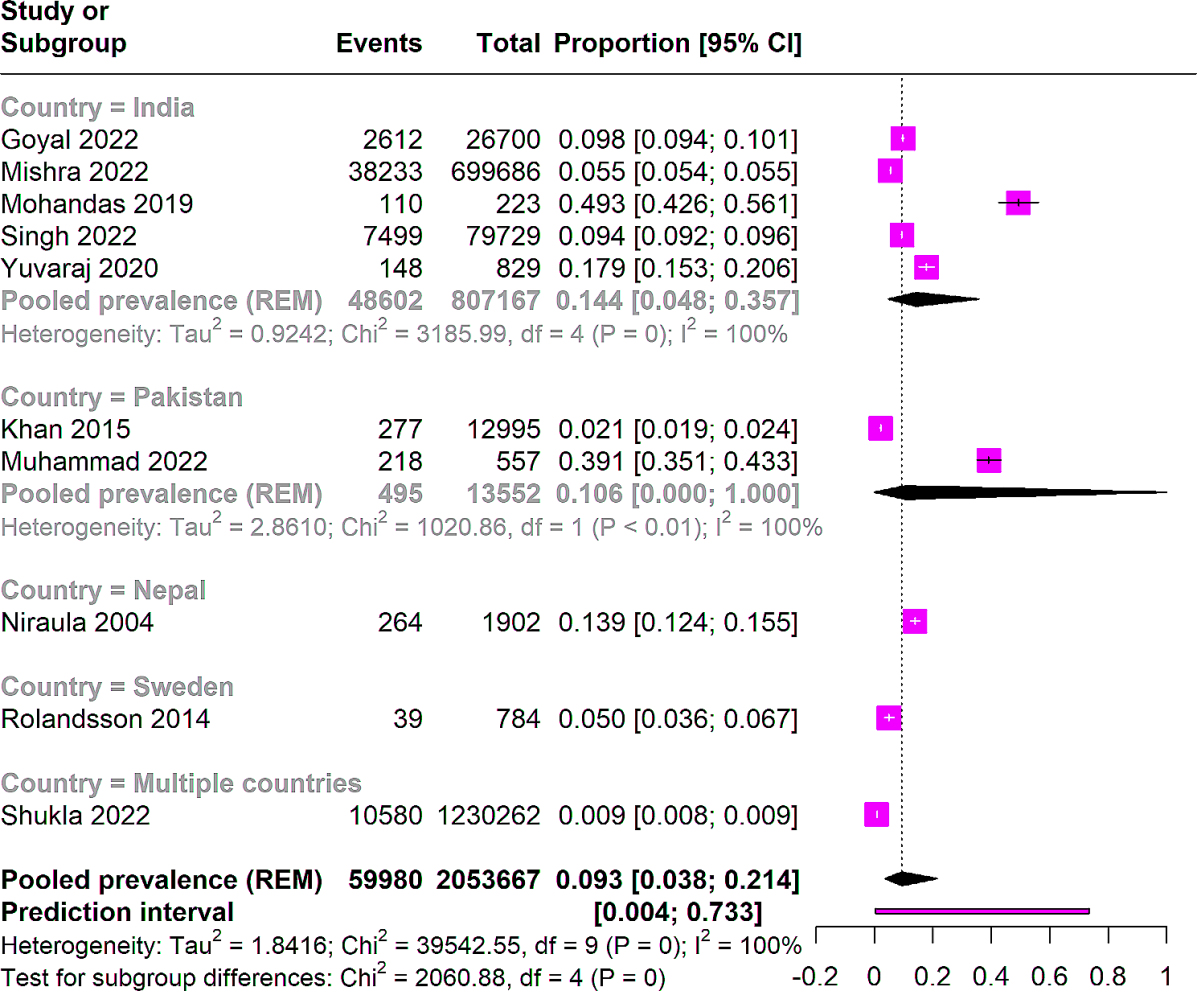
Subgroup analysis of SLT use among women of reproductive age based on country

**Fig. 5:**
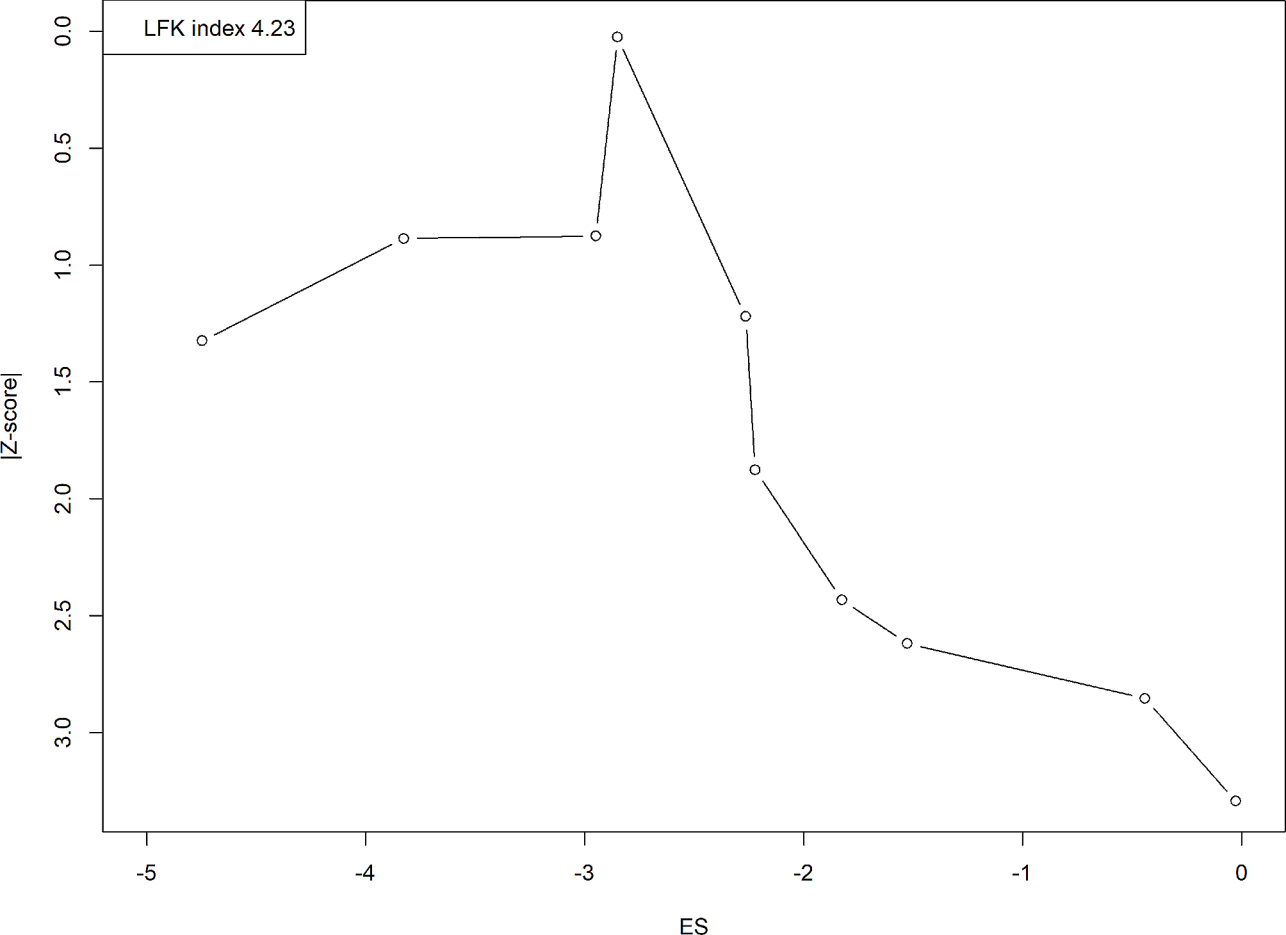
Doi plot illustrating the publication bias

We performed a sensitivity analysis by leaving one study out at a time. No significant change was observed in the overall analysis. Omitting the study by Mohandas et al. reduced the prevalence to 7.4%. Similarly, omitting Shukla et al. increased the prevalence to 12% (Fig. 4). In assessing the potential for publication bias within our systematic review, we utilized a Doi plot accompanied by the LFK index. The Doi plot revealed an evident asymmetry in the distribution of the effect sizes, which were plotted against their respective z-scores. The majority of studies clustered to one side of the effect size spectrum, which is a visual indicator of potential publication bias. This visual indication was quantitatively supported by an LFK index of 4.23, significantly surpassing the threshold of 1, which is typically used to denote substantial asymmetry. This high LFK index suggests that our meta-analysis may be influenced by publication bias, where studies with non-significant or negative results could be underrepresented in the literature. The implications of this are critical; the effect sizes reported in our review should be interpreted with caution, as the true effects could be overestimated due to the preferential publication of studies with positive results (Fig. 5).

## Discussion

To our knowledge, this study is the first systematic review and meta-analysis to assess the prevalence of SLT use among women of reproductive age. Our findings indicate a considerable prevalence of SLT use in this demographic, which has significant public health implications. The pooled prevalence of 9% necessitate the need for targeted interventions and policy reforms, especially in regions with higher usage rates.

The nature of included studies varied in our review. The study by Rolandsson et al. [54] is the only analysis conducted in a developed country, Sweden, focusing on female athletes. It revealed that a vast majority of the participants had never smoked or used snuff, highlighting the healthy behaviors of this population. However, a notable number had experimented with snus (20%) or smoking (27%), though the regular use of snus was comparatively low against other countries reviewed. This may reflect better awareness of tobacco’s negative impacts and heightened health consciousness in Sweden. The study also found significant correlations between the smoking behaviors of female athletes and those of their mothers and peers. Another study by Khan et al. presents significant insights into the use of alternative tobacco products, such as chewing tobacco (CT), among women of reproductive age in Pakistan [51]. The prevalence of these alternative tobacco forms aligns with the most recent national data reported by the World Health Organization. Notably, there’s a higher usage rate among vulnerable groups of Pakistani women in their reproductive years. Several factors must be considered when analyzing this prevalence. While the overall tobacco use among women is less than that of men, factors such as aggressive marketing, the rising popularity of alternative tobacco forms like waterpipe, and changing societal attitudes towards women can increase their susceptibility to tobacco-related risks. The study also highlights that women with a lower socio-economic status (no education or poor) are more prone to using alternative tobacco forms. This trend is particularly evident among the poor, uneducated, rural inhabitants, and those who have not accessed health facilities in the last year, indicating a higher propensity for health risk behaviors like CT use [51]. Mishra and colleagues discovered a significant link between the use of tobacco products, including smoking and smokeless forms, as well as alcohol consumption, and an increased incidence of Non-Communicable Diseases (NCDs) in women [46].

Similar findings highlighting the issue of SLT were also reported in other studies. Particularly, a known risk factor for oral cancers, contributing to morbidity in Indian women, especially those from lower socioeconomic backgrounds [55–58]. The combined effects of tobacco and alcohol use were found to significantly elevate the prevalence of NCDs in women. This study underscores the substantial population-attributable risk posed by smoking, using SLT, and alcohol consumption for NCDs among women. These findings align with previous research from India and other developing countries, indicating a heightened risk of hypertension and other NCDs due to smoking and alcohol use. Consequently, these results underscore the urgent need to develop effective prevention strategies to counter the rising trend of NCDs by addressing tobacco and alcohol consumption. Similarly, Muhammad et al. proposed that gutka (chewable SLT mixture consisting of betel nut and catechu) use is a significant predictor of anemia, a finding that might hold true for other coastal slums as well [50]. The study posits that gutka’s components could interfere with intestinal iron absorption and disrupt various biological processes. Notably, chewable tobacco, a key ingredient in gutka, contains iron that impacts iron metabolism, hemoglobin levels, and iron stores. This could potentially influence or diminish the expression of hepcidin mRNA and lower hepcidin levels, which are essential for maintaining iron balance, especially in pregnant women [59–62]. Furthermore, the crushed areca nut in gutka, which includes alkaloids, may impair the intestine’s ability to absorb iron. Additionally, calcium hydroxide, commonly found in gutka, is known to inhibit iron absorption [63]. Studies suggests that the observed link between gutka use and anemia might be due to these mechanisms [50, 64–66]. Its effect is profound even in non-pregnant women. SLT can lead to impairments in ovarian function, morphology, oocyte quality, and hormonal regulation [28].

The implication of our findings are profound. Effective public health interventions need to be targeted at regions with higher SLT usage. These interventions should focus on education about the risks associated with SLT. Addressing socioeconomic disparities is crucial, involving educational programs and support services in rural and impoverished areas to assist women in quitting tobacco use. Furthermore, stricter regulation of the marketing and accessibility of alternative tobacco products, such as chewable tobacco, is necessary to reduce consumption. Integrating tobacco cessation programs into routine healthcare services, especially for women, can reduce tobacco use and promote healthier behaviors. Policies should also concurrently address the compounded effects of tobacco and alcohol use of NCDs. There is a need for longitudinal research to understand evolving tobacco consumption behaviors among women, considering changing societal attitudes and marketing strategies. Qualitative research exploring the cultural and social reasons behind tobacco use among women can provide crucial insights for developing culturally sensitive interventions. Additionally, more high-quality studies with large sample size from different geographical location are required. More studies are needed to investigate the relationship between healthcare access and tobacco use, and how healthcare interventions impact tobacco use behaviors. Finally, assessing the efficacy of different public health interventions and policies aimed at reducing SLT use among women is essential, including studies on the impact of educational campaigns, healthcare integration, and regulatory changes.

Our study has some limitations. One primary limitation is the restriction to articles published only in English. We searched only four databases and did not consider grey literature and non-indexed journals in our search. The sampling methods of the included studies varied, which may affect the results. Additionally, the scarcity of research specifically focusing on this demographic limit our ability to draw broad conclusions, as the number of studies exclusively examining SLT use among women of reproductive age is quite limited. Furthermore, the presence of publication bias in our study is an inevitable factor, where studies with more significant or higher prevalence rates are more likely to be published. This bias could lead to an overestimation of the prevalence of SLT use and underscores the need for more comprehensive research. Future research should delve into cultural and social factors influencing tobacco use among women, the interaction between healthcare access and tobacco habits, and the impact of specific public health interventions. Comprehensive studies are required to provide a deeper understanding of SLT use patterns and develop culturally tailored intervention programs. Research into how access to healthcare and specific healthcare interventions affect tobacco behavior is vital. Evaluating the effectiveness of various public health strategies, including educational, healthcare, and regulatory measures, is necessary to identify the most impactful approaches to reducing SLT use among women.

## Conclusion

The study shows a significant prevalence of SLT use in women of reproductive age, especially in low socioeconomic and developing countries like India, Pakistan, and Nepal. For women of reproductive age, the use of SLT introduces additional concerns, such as infertility, complications during pregnancy, and adverse fetal outcomes, such as low birth weight and preterm birth. The results highlight the necessity for specific public health measures and policy changes to decrease SLT consumption among reproductive-age women. Further studies are needed to investigate the reasons behind SLT usage in this group and to assess the impact of intervention strategies, with the goal of guiding more effective public health initiatives and policies.

### Electronic supplementary material

Below is the link to the electronic supplementary material.

Supplementary Material 1

## Data Availability

No datasets were generated or analysed during the current study.
