# Supplementary material for: Smokeless tobacco consumption among women of reproductive age: a systematic review and meta-analysis
Source: BMC Public Health. 2024 May 20;24:1361. doi: 10.1186/s12889-024-18840-z (PMC11106917; doi:10.1186/s12889-024-18840-z)
Supplement: Supplementary file 1 — Supplementary Material 1 [file 12889_2024_18840_MOESM1_ESM.docx]

**SUPPLEMENTARY MATERIALS**

**Table S1.** PRISMA Checklist

| **Section and Topic** | **Item #** | **Checklist item (Smokeless tobacco consumption among women of reproductive age: A systematic review and meta-analysis)** | **Location where item is reported** |
| --- | --- | --- | --- |
| **TITLE** | | |  |
| Title | 1 | Identify the report as a systematic review. | 1 |
| **ABSTRACT** | | |  |
| Abstract | 2 | See the PRISMA 2020 for Abstracts checklist. (made as per the Journal guidelines) | 2 |
| **INTRODUCTION** | | |  |
| Rationale | 3 | Describe the rationale for the review in the context of existing knowledge. | 3 |
| Objectives | 4 | Provide an explicit statement of the objective(s) or question(s) the review addresses. | 3 |
| **METHODS** | | |  |
| Eligibility criteria | 5 | Specify the inclusion and exclusion criteria for the review and how studies were grouped for the syntheses. | 3 |
| Information sources | 6 | Specify all databases, registers, websites, organisations, reference lists and other sources searched or consulted to identify studies. Specify the date when each source was last searched or consulted. | 3 |
| Search strategy | 7 | Present the full search strategies for all databases, registers and websites, including any filters and limits used. | Table S2 |
| Selection process | 8 | Specify the methods used to decide whether a study met the inclusion criteria of the review, including how many reviewers screened each record and each report retrieved, whether they worked independently, and if applicable, details of automation tools used in the process. | 4 |
| Data collection process | 9 | Specify the methods used to collect data from reports, including how many reviewers collected data from each report, whether they worked independently, any processes for obtaining or confirming data from study investigators, and if applicable, details of automation tools used in the process. | 4 |
| Data items | 10a | List and define all outcomes for which data were sought. Specify whether all results that were compatible with each outcome domain in each study were sought (e.g., for all measures, time points, analyses), and if not, the methods used to decide which results to collect. | 4, Table 1 |
|  | 10b | List and define all other variables for which data were sought (e.g., participant and intervention characteristics, funding sources). Describe any assumptions made about any missing or unclear information. | 4 |
| Study risk of bias assessment | 11 | Specify the methods used to assess risk of bias in the included studies, including details of the tool(s) used, how many reviewers assessed each study and whether they worked independently, and if applicable, details of automation tools used in the process. | 4, |
| Effect measures | 12 | Specify for each outcome the effect measure(s) (e.g. risk ratio, mean difference) used in the synthesis or presentation of results. | 5 |
| Synthesis methods | 13a | Describe the processes used to decide which studies were eligible for each synthesis (e.g. tabulating the study intervention characteristics and comparing against the planned groups for each synthesis (item #5)). | 5, Table 1 |
|  | 13b | Describe any methods required to prepare the data for presentation or synthesis, such as handling of missing summary statistics, or data conversions. | NA |
|  | 13c | Describe any methods used to tabulate or visually display results of individual studies and syntheses. | 5 |
|  | 13d | Describe any methods used to synthesize results and provide a rationale for the choice(s). If meta-analysis was performed, describe the model(s), method(s) to identify the presence and extent of statistical heterogeneity, and software package(s) used. | 5 |
|  | 13e | Describe any methods used to explore possible causes of heterogeneity among study results (e.g. subgroup analysis, meta-regression). | 5 |
|  | 13f | Describe any sensitivity analyses conducted to assess robustness of the synthesized results. | 5 |
| Reporting bias assessment | 14 | Describe any methods used to assess risk of bias due to missing results in a synthesis (arising from reporting biases). | NA |
| Certainty assessment | 15 | Describe any methods used to assess certainty (or confidence) in the body of evidence for an outcome. | NA |
| **RESULTS** | | |  |
| Study selection | 16a | Describe the results of the search and selection process, from the number of records identified in the search to the number of studies included in the review, ideally using a flow diagram. | 5, Figure-1, 2 |
|  | 16b | Cite studies that might appear to meet the inclusion criteria, but which were excluded, and explain why they were excluded. | 5, Table 1 |
| Study characteristics | 17 | Cite each included study and present its characteristics. | Table-1 |
| Risk of bias in studies | 18 | Present assessments of risk of bias for each included study. | Table S4 |
| Results of individual studies | 19 | For all outcomes, present, for each study: (a) summary statistics for each group (where appropriate) and (b) an effect estimate and its precision (e.g. confidence/credible interval), ideally using structured tables or plots. | Table 1, Figure 2, 3 |
| Results of syntheses | 20a | For each synthesis, briefly summarise the characteristics and risk of bias among contributing studies. | 5 |
|  | 20b | Present results of all statistical syntheses conducted. If meta-analysis was done, present for each the summary estimate and its precision (e.g. confidence/credible interval) and measures of statistical heterogeneity. If comparing groups, describe the direction of the effect. | 5, 6 Figure 2, 3 |
|  | 20c | Present results of all investigations of possible causes of heterogeneity among study results. | 5, 6 |
|  | 20d | Present results of all sensitivity analyses conducted to assess the robustness of the synthesized results. | NA |
| Reporting biases | 21 | Present assessments of risk of bias due to missing results (arising from reporting biases) for each synthesis assessed. | NA |
| Certainty of evidence | 22 | Present assessments of certainty (or confidence) in the body of evidence for each outcome assessed. | NA |
| **DISCUSSION** | | |  |
| Discussion | 23a | Provide a general interpretation of the results in the context of other evidence. | 6 |
|  | 23b | Discuss any limitations of the evidence included in the review. | 7 |
|  | 23c | Discuss any limitations of the review processes used. | 7 |
|  | 23d | Discuss implications of the results for practice, policy, and future research. | 7 |
| **OTHER INFORMATION** | | |  |
| Registration and protocol | 24a | Provide registration information for the review, including register name and registration number, or state that the review was not registered. | 4 |
|  | 24b | Indicate where the review protocol can be accessed, or state that a protocol was not prepared. | 4 |
|  | 24c | Describe and explain any amendments to information provided at registration or in the protocol. | NA |
| Support | 25 | Describe sources of financial or non-financial support for the review, and the role of the funders or sponsors in the review. | 8 |
| Competing interests | 26 | Declare any competing interests of review authors. | 8 |
| Availability of data, code and other materials | 27 | Report which of the following are publicly available and where they can be found: template data collection forms; data extracted from included studies; data used for all analyses; analytic code; any other materials used in the review. | 8 |

**Table S2.** Inclusion and Exclusion criteria

**Research Question: “What is the pooled prevalence of smokeless tobacco use in reproductive-aged women?”**

| **Inclusion** | | **Exclusion** |
| --- | --- | --- |
| **Participants** | - Only women of reproductive age (15 to 49). |  |
| **Intervention/Exposure** | - Smokeless or chewing tobacco |  |
| **Outcome** | - No. of Smokeless or chewing tobacco users in total sample (prevalence) |  |
| **Study Designs** | Cross-sectional, trials and cohort studies | Letter to editor,  Commentaries,  Qualitative studies, Abstract only, Case series, case reports, reviews, Discussion papers |
|  | Only published articles with English language restriction till 11^th^ of November 2023 | Unavailable full-text articles |

**Table S3.** The adjusted search terms as per searched electronic databases [as of 11.11.2023]

| Database | Search Query | Results |
| --- | --- | --- |
|  | | |
| PubMed | **((("smokeless"[All Fields]) AND ("tobacco"[MeSH Terms] OR "tobacco"[All Fields] OR "tobacco products"[MeSH Terms] OR ("tobacco"[All Fields] AND "products"[All Fields]) OR "tobacco products"[All Fields] OR "tobaccos"[All Fields] OR "tobacco s"[All Fields])) OR "Chewing tobacco"[All Fields] OR ("tobacco, smokeless"[MeSH Terms] OR ("tobacco"[All Fields] AND "smokeless"[All Fields]) OR "smokeless tobacco"[All Fields] OR "snuff"[All Fields] OR "snuffs"[All Fields]) OR ("tobacco, smokeless"[MeSH Terms] OR ("tobacco"[All Fields] AND "smokeless"[All Fields]) OR "smokeless tobacco"[All Fields] OR "snus"[All Fields]) OR "Khaini"[All Fields] OR "Gutkha"[All Fields] OR "Naswar"[All Fields] OR "Tobacco pouches"[All Fields] OR "Spit tobacco"[All Fields] OR "Oral tobacco"[All Fields] OR "chewed tobacco"[All Fields]) AND ("Reproductive age"[All Fields] OR ("childbearers"[All Fields] OR "childbearing"[All Fields]) OR "Fertile years"[All Fields] OR "Age of fertility"[All Fields] OR "young women"[All Fields])–** | **34** |
|  | | |
| Embase | (smokeless AND tobacco OR 'chewing tobacco' OR snuff OR snus OR khaini OR gutkha OR naswar OR 'tobacco pouches' OR 'spit tobacco' OR 'oral tobacco' OR 'chewed tobacco') AND ('reproductive age' OR childbearing OR 'fertile years' OR 'age of fertility' OR 'young women') | 44 |
|  | | |
| Web of Science | ((smokeless AND tobacco) OR "Chewing tobacco" OR Snuff OR Snus OR khaing OR gurkha OR nascar OR "Tobacco pouches" OR "Spit tobacco" OR "Oral tobacco" OR "chewed tobacco") (All Fields) and ("Reproductive age" OR Childbearing OR "Fertile years" OR "Age of fertility" OR "young women") (All Fields) | 32 |
|  | |  |
| Scopus | ( TITLE-ABS-KEY ( ( "Reproductive age" OR childbearing OR "Fertile years" OR "Age of fertility" OR "young women" ) ) AND TITLE-ABS-KEY ( ( ( smokeless AND tobacco ) OR "Chewing tobacco" OR snuff OR snus OR khaini OR gutkha OR naswar OR "Tobacco pouches" OR "Spit tobacco" OR "Oral tobacco" OR "chewed tobacco" ) ) ) | 45 |

**Table S4.** Quality assessment using a JBI quality assessment tool for prevalence studies.

| Study | D1 | D2 | D3 | D4 | D5 | D6 | D7 | D8 | D9 | Overall Quality of the study |
| --- | --- | --- | --- | --- | --- | --- | --- | --- | --- | --- |
|  | Was the sample frame appropriate to address the target population? | Were study participants sampled in an appropriate way? | Was the sample size adequate? | Were the study subjects and the setting described in detail? | Was the data analysis conducted with sufficient coverage of the identified sample? | Were valid methods used for the identification of the condition? | Was the condition measured in a standard, reliable way for all participants? | Was there appropriate statistical analysis? | Was the response rate adequate, and if not, was the low response rate managed appropriately? |  |
| Goyal et al. 2022 | Yes | Yes | Yes | Yes | Unclear | Yes | Unclear | Yes | Unclear | Moderate |
| Khan et al. 2015 | Yes | Yes | Yes | Yes | Yes | NA | Yes | Yes | Yes | High |
| Mishra et al. 2022 | Yes | Yes | Yes | Yes | Yes | Yes | Unclear | Yes | Unclear | Moderate |
| Mohandas et al. 2019 | Yes | No | Yes | Yes | Yes | Yes | Yes | Yes | Unclear | Moderate |
| Muhammad et al. 2022 | Yes | Yes | No | Yes | Yes | Yes | Yes | Unclear | Unclear | Moderate |
| Niraula et al. 2004 | Yes | Yes | Yes | Yes | Yes | Yes | Yes | Yes | Unclear | High |
| Rolandsson et al. 2014 | Yes | Yes | Yes | Yes | Unclear | Yes | Yes | Yes | Unclear | Moderate |
| Shukla et al. 2021 | Yes | Yes | Yes | Yes | Yes | Yes | Yes | Yes | Unclear | High |
| Singh et al. 2022 | Yes | Yes | Yes | Yes | Unclear | Yes | Yes | Yes | Unclear | Moderate |
| Yuvaraj et al. 2020 | Yes | No | Yes | Yes | Yes | Unclear | Yes | Yes | Yes | Moderate |
